# Supplementary material for: A Micro Electrochemical Sensor for Multi-Analyte Detection Based on Oxygenated Graphene Modified Screen-Printed Electrode
Source: Nanomaterials (Basel). 2022 Feb 21;12(4):711. doi: 10.3390/nano12040711 (PMC8875984; doi:10.3390/nano12040711)
Supplement: Supplementary file 1 [file nanomaterials-12-00711-s001.zip › nanomaterials-1585096-supplementary.pdf]

Supplementary Material

A Micro Electrochemical Sensor for Multi-Analyte Detection Based on Oxygenated Graphene Modified Screen-Printed Electrode

Baiqing Yuan *, Liju Gan, Gang Li, Chunying Xu and Gang Liu *

School of Chemistry and Materials Science, Ludong University, Yantai 264025, China; gan17737233930@163.com (L.G.); 1258331922@126.com (G.L.); chunyingxu@126.com (C.X.)

* Correspondence: baiqingyuan1981@126.com & bqyuan@ldu.edu.cn (B.Y.); shdliugang@163.com (G.L.)

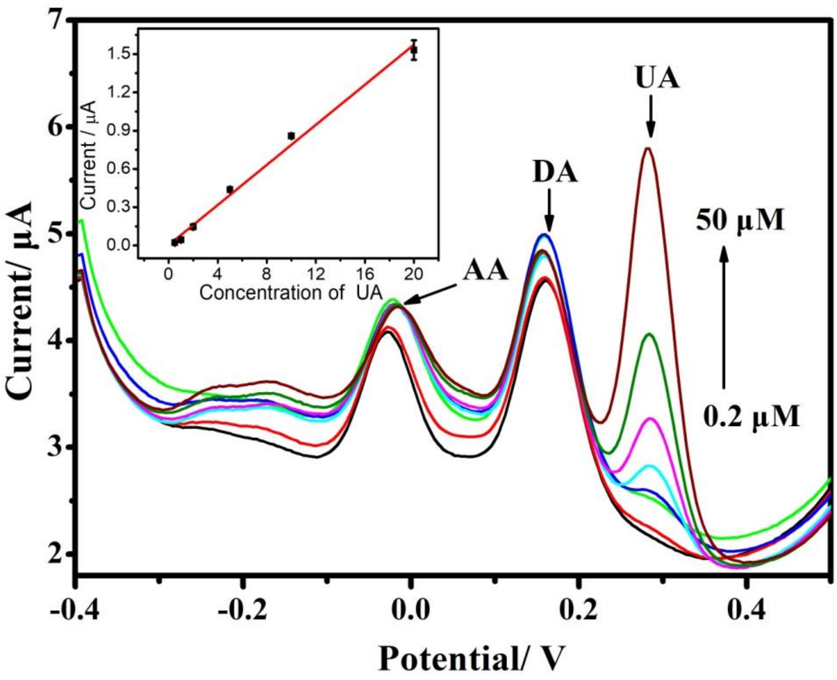

Figure S1 Electrochemical detection of UA in the presence of AA and DA at $GO_{-0.75\ V}$/GCE in 0.1 M pH 7.2 PBS. Inset: calibration plot for the peak current versus UA concentrations. Conditions: Inc E, 4 mV; Amplitude, 0.05V; Pulse width, 0.2 s; Sample width, 0.0167 s; Pulse period, 0.5 s.

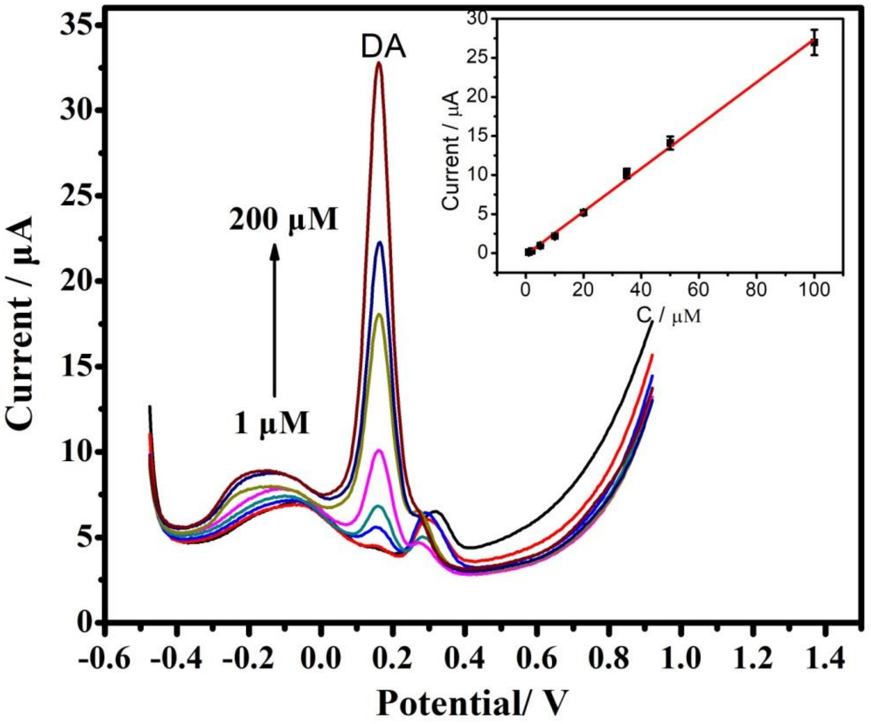

Figure S2 Electrochemical detection of DA in the presence of AA and UA at GO-0.75 V/GCE in 0.1 M pH 7.2 PBS. Inset: calibration plot for the peak current versus DA concentrations. Conditions are the same as in Figure S1.

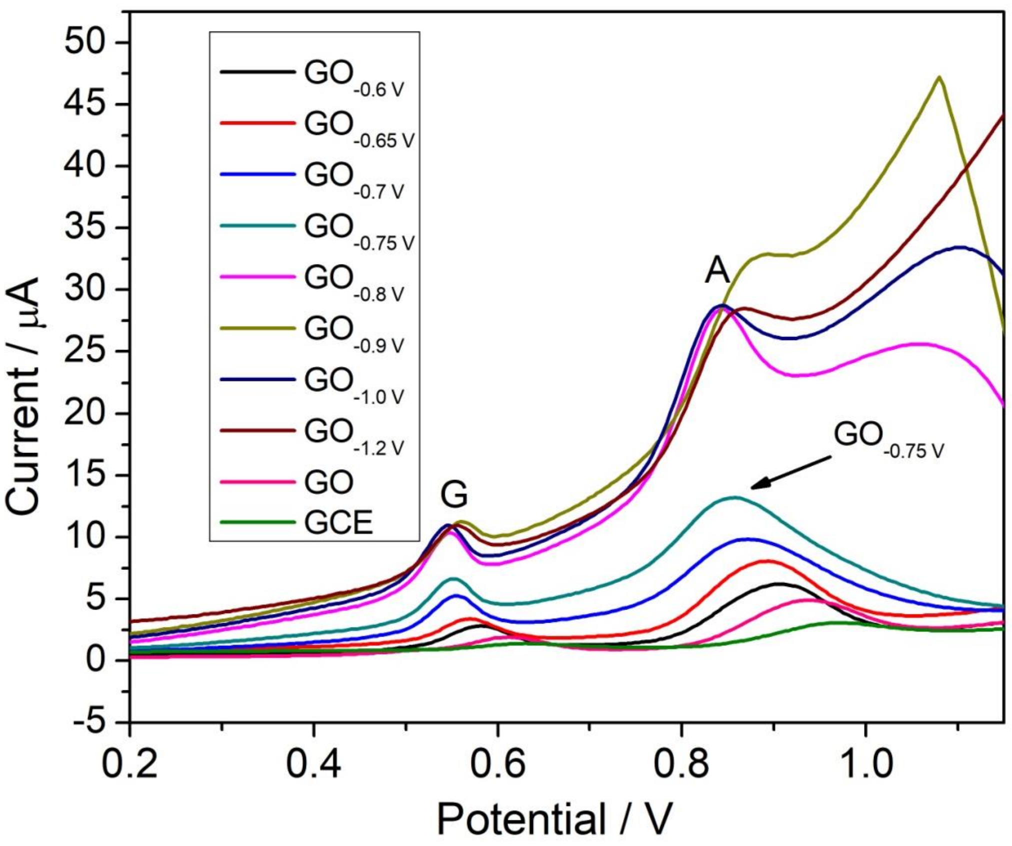

Figure S3 DPV of the mixture of G and A in 0.1 M pH 7.2 PBS at the GCE, GO, and GO/GCE electrochemically treated at different potentials. Conditions are the same as in Figure S1.

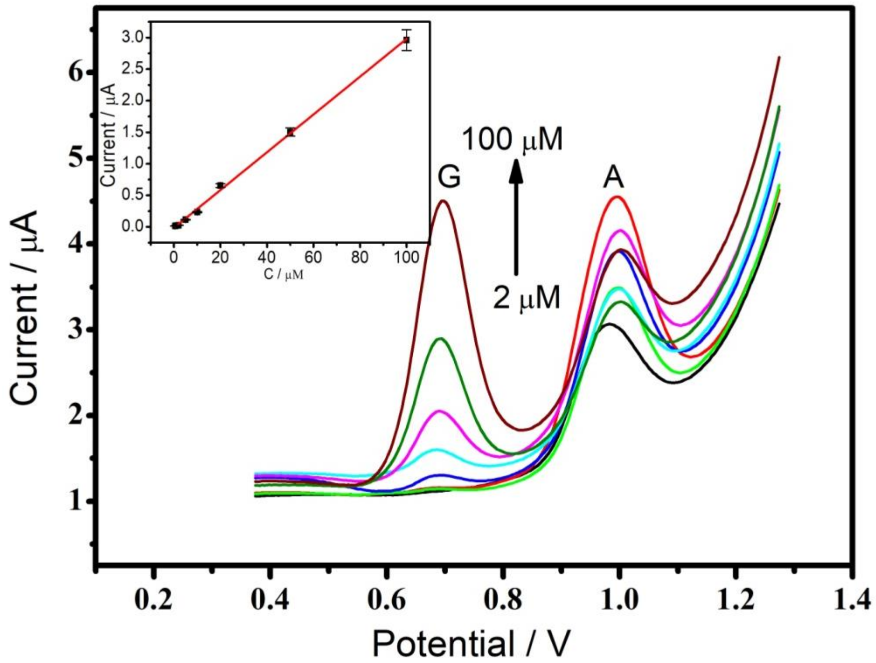

Figure S4 Electrochemical detection of G in the presence of A at GO$_{-0.75}$ V/GCE in 0.1 M pH 7.2 PBS. Inset: calibration plot for the peak current versus G concentrations. Conditions are the same as in Figure S1.

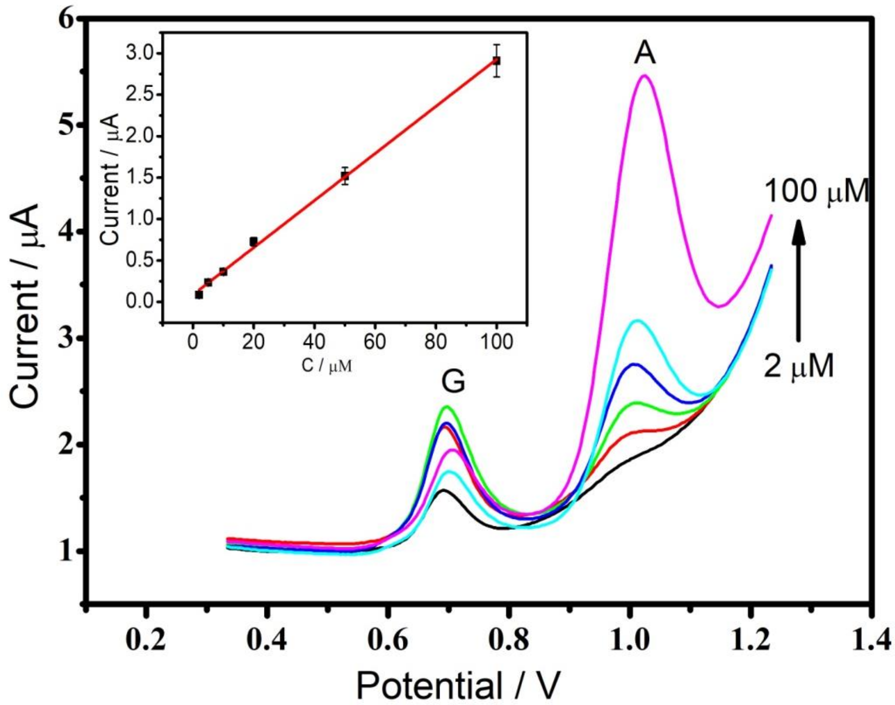

Figure S5 Electrochemical detection of A in the presence of G at GO$_{-0.75}$ V/GCE in 0.1 M pH 7.2 PBS. Inset: calibration plot for the peak current versus A concentrations. Conditions are the same as in Figure S1.

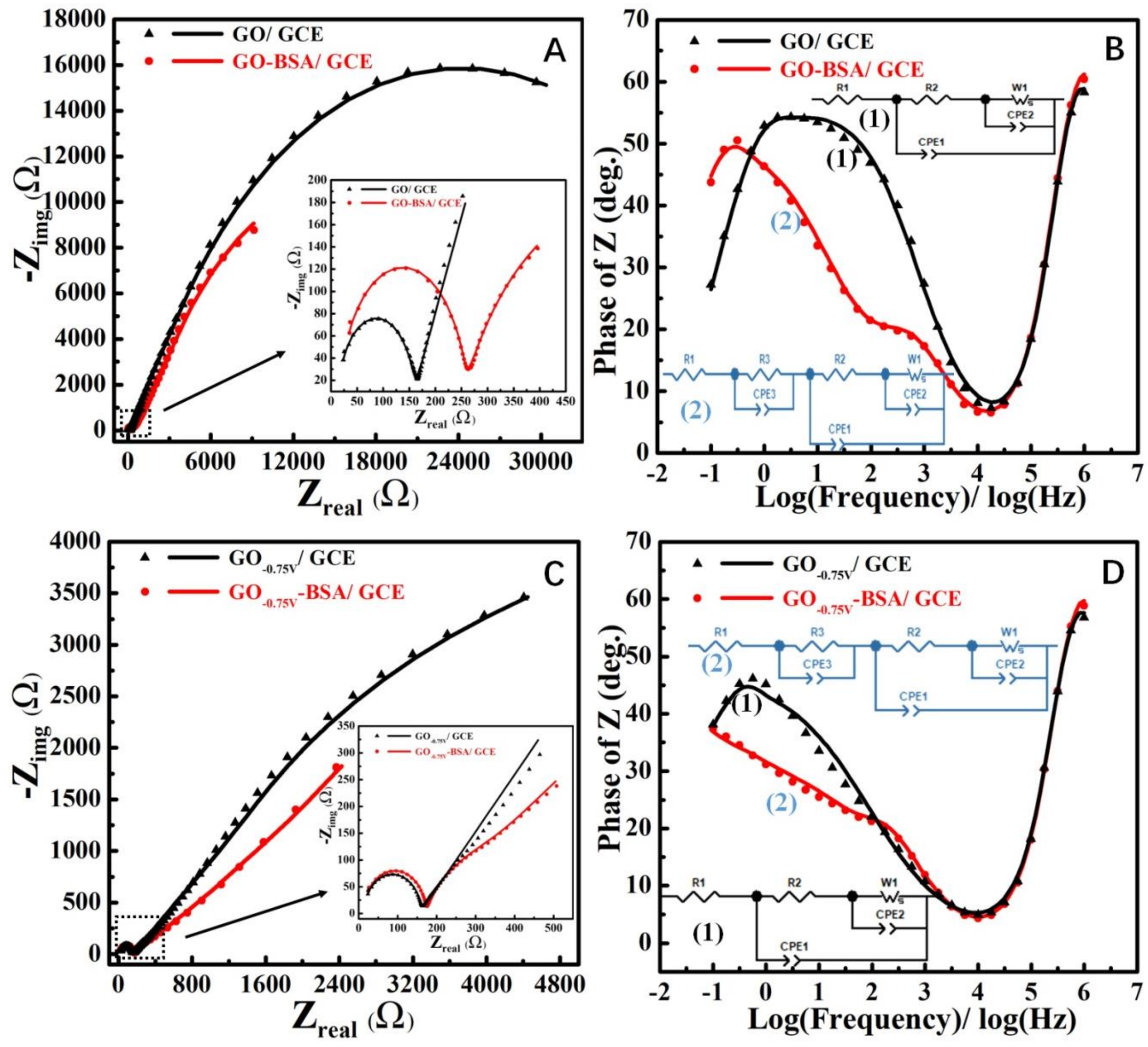

Figure S6 Nyquist diagrams (A and C) and phase angle diagrams vs log of frequency of Bode plots (B and D) for different electrodes. The symbols and solid lines present the experimental and the fitted data, respectively. The insets in phase angle diagrams are the corresponding electrical equivalent circuits. EIS was carried out in 0.1 M KCl containing 5 mM $Fe(CN)_6{}^{3-/4-}$ in the frequency range from 1 MHz to 0.1 Hz at 0.24 V.

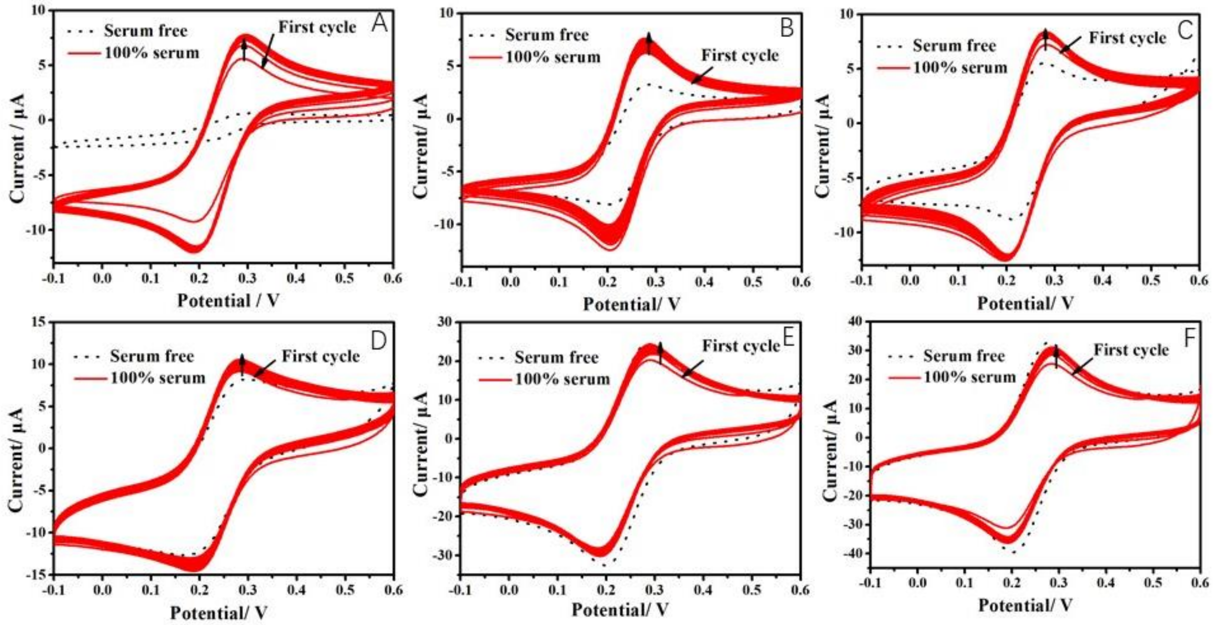

Figure S7 CVs of 5 mM Fe(CN)6 3-/4- redox couple at different GOpotential /GCE (dotted line) and GOpotential-serum /GCE (solid line) at the scan rate of 25 mV/s. A: GO, B: GO-0.6 V, C: GO-0.65 V, D: GO-0.75 V, E:GO-0.85 V, F:GO-0.95 V.

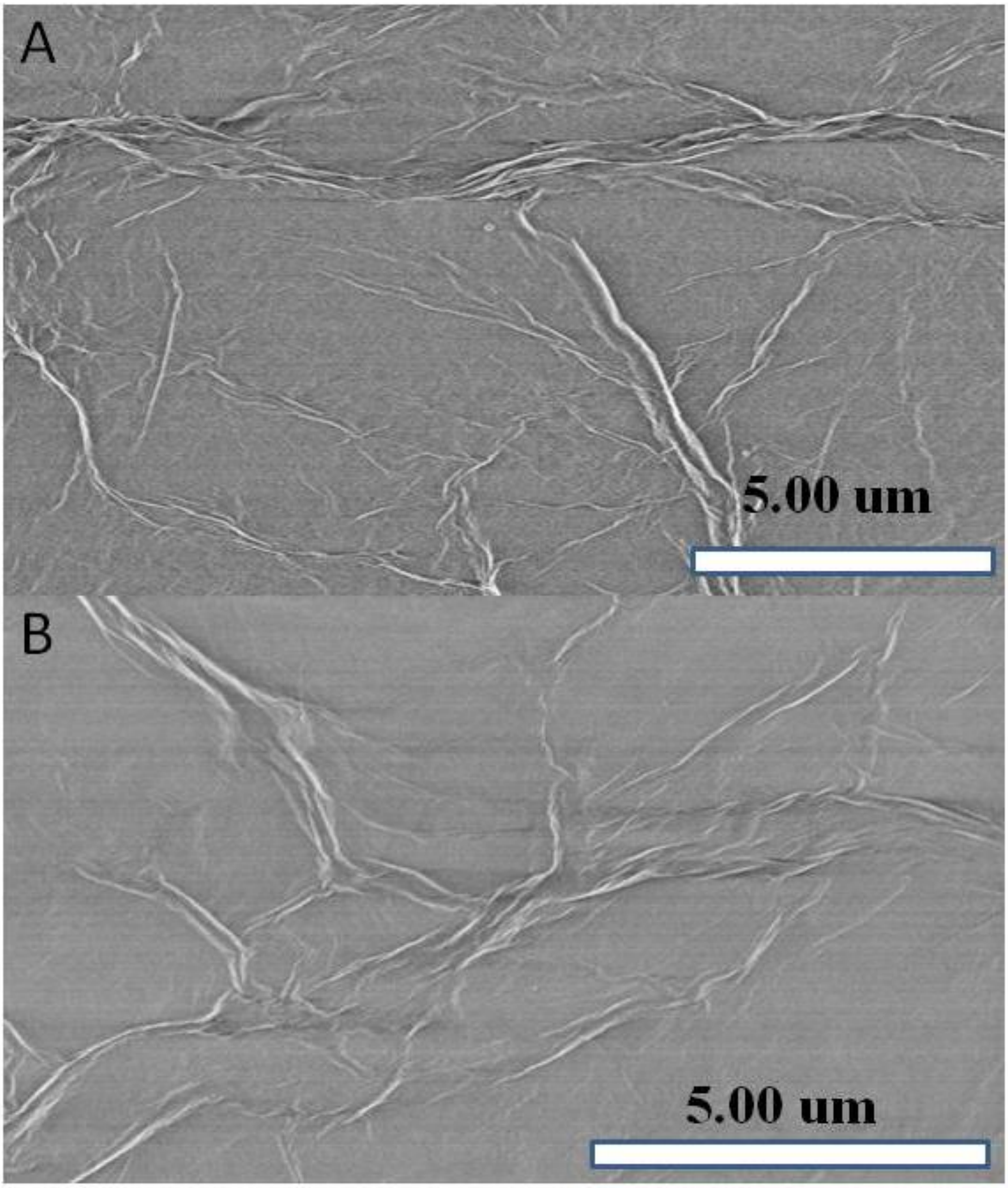

Figure S8 SEM images of $GO_{-0.75\ V}$/GCE (A) and $GO_{-0.75\ V}$-BSA/GCE (B).

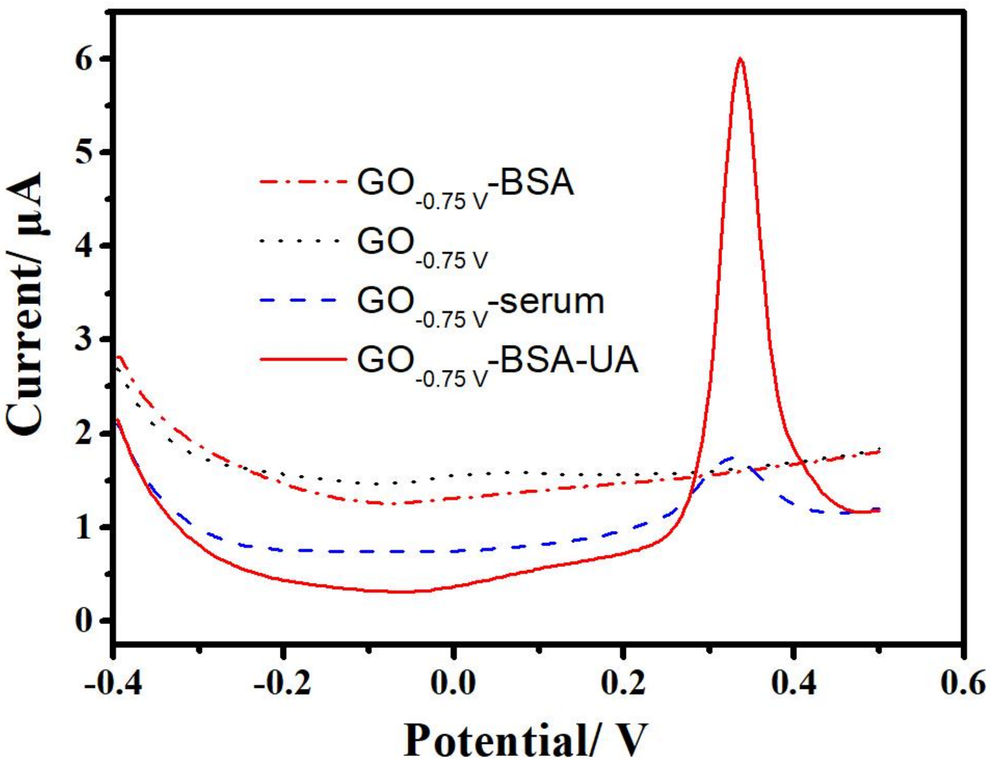

Figure S9 DPV of $GO_{-0.75\ V}$/GCE, $GO_{-0.75\ V}$-BSA/GCE, $GO_{-0.75\ V}$-serum/GCE and $GO_{-0.75\ V}$-BSA-UA/GCE in background electrolyte (0.1 M pH 7.2 PBS). Conditions are the same as in Figure S1.

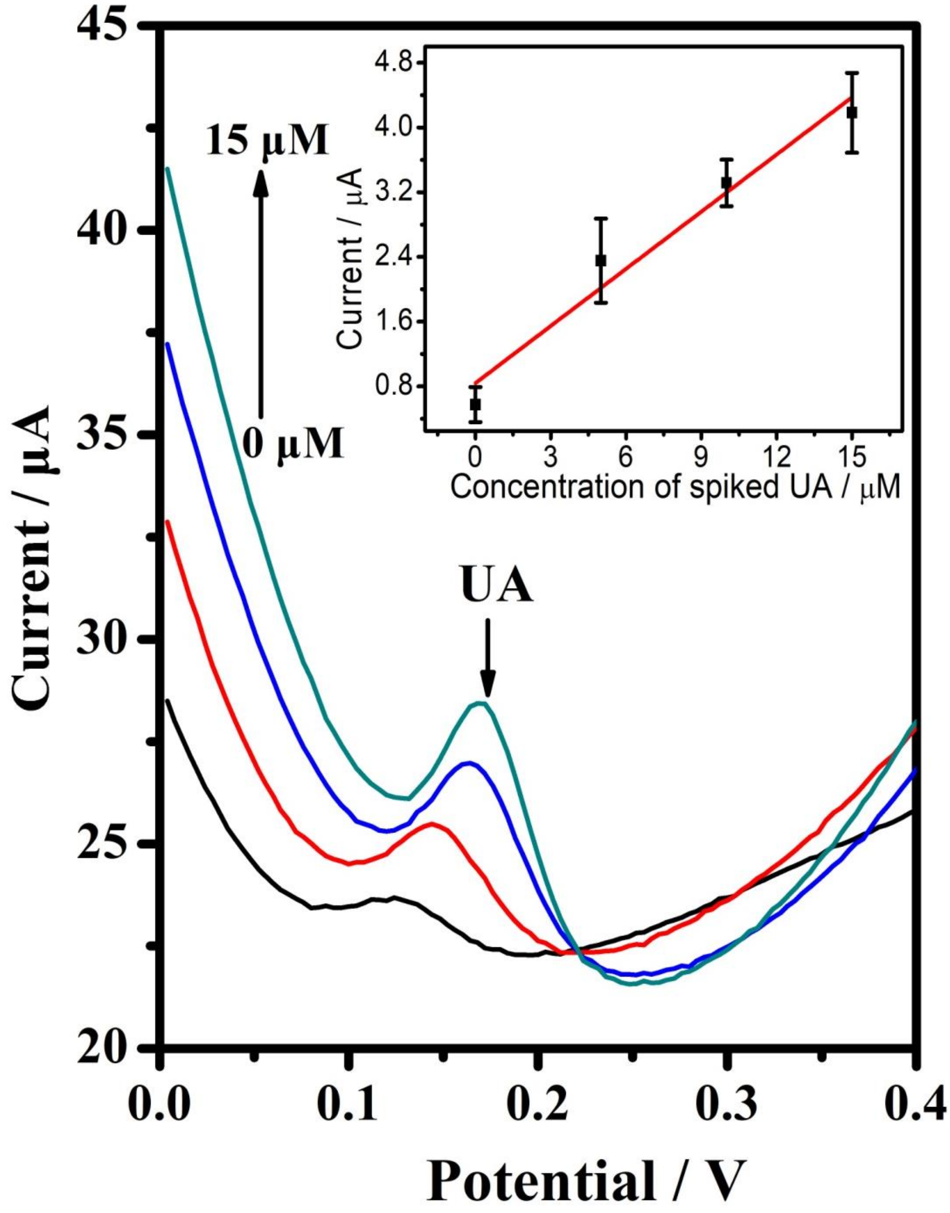

Figure S10 Standard addition method for the measurement of UA in 40-folds diluted serum. Conditions are the same as in Figure S1.

Table S1 Obtained parameters from fitting for different electrodes.

Electrodes	R1	R2	R3	W1-R	W1-T	W1-P	CPE1-T	CPE1-P	CPE2-T	CPE2-P	CPE3-T	CPE3-P
GO/GCE	14.57	142.5		48024	0.71293	0.44544	2.273E-9	1.036	1.544E-5	0.70518		
GO-BSA/GCE	17.9	235.8	220.2	38392	3.144	0.49568	2.004E-9	1.012	6.5695E-5	0.63353	1.0947E-5	0.77777
$GO_{-0.75V}$/GCE	13.99	142.6		18666	1.854	0.52165	3.375E-9	1.012	1.6222 E-4	0.51987		
$GO_{-0.75V}$-BSA/GCE	12.74	158.1	259.2	8902	22.03	0.45293	3.459E-9	1.003	1.0976E-5	1.181	3.4925E-5	0.727
